# Weight-loss in obese dogs promotes important shifts in fecal microbiota profile to the extent of resembling microbiota of lean dogs

**DOI:** 10.1186/s42523-021-00160-x

**Published:** 2022-01-06

**Authors:** Henrique Tobaro Macedo, Mariana Fragoso Rentas, Thiago Henrique Annibale Vendramini, Matheus Vinicius Macegoza, Andressa Rodrigues Amaral, Juliana Toloi Jeremias, Júlio César de Carvalho Balieiro, Karina Pfrimer, Eduardo Ferriolli, Cristiana Ferreira Fonseca Pontieri, Marcio Antonio Brunetto

**Affiliations:** 1 Department of Animal Nutrition and Production, Pet Nutrology Research Center (CEPEN Pet), School of Veterinary Medicine and Animal Science (FMVZ), University of São Paulo (USP), Duque de Caxias Norte Ave, 255, Pirassununga, São Paulo 13635-900 Brazil; 2 Nutritional Development Center (CDN), Grandfood Indústria E Comércio LTDA (Premier Pet), Luiz Augusto de Oliveira Hwy, Km 204, Dourado, São Paulo 13590-000 Brazil; 3 Medical School of Ribeirão Preto (FMRP), University of São Paulo (USP), Bandeirantes Ave, 3900 - Campus da USP, Ribeirão Preto, São Paulo 14049-900 Brazil

**Keywords:** Canine, Dysbiosis, Gastrointestinal tract, Microbiome, Obesity

## Abstract

**Background:**

Among the undesirable changes associated with obesity, one possibility recently raised is dysbiosis of the intestinal microbiota. Studies have shown changes in microbiota in obese rats and humans, but there are still few studies that characterize and compare the fecal microbiota of lean, obese and dogs after weight loss. Thus, this study aimed to evaluate the effects of a weight loss program (WLP) in fecal microbiota of dogs in addition to comparing them with those of lean dogs. Twenty female dogs of different breeds, aged between 1 and 9 years were selected. They were equally divided into two groups: Obese group (OG), with body condition score (BCS) 8 or 9/9, and body fat percentage greater than 30%, determined by the deuterium isotope dilution method, and lean group (LG) with BCS 5/9, and maximum body fat of 15%. Weight loss group (WLG) was composed by OG after loss of 20% of their current body weight. Fecal samples were collected from the three experimental groups. Total DNA was extracted from the feces and these were sequenced by the Illumina methodology. The observed abundances were evaluated using a generalized linear model, considering binomial distribution and using the logit link function in SAS (*p* < 0.05).

**Results:**

The WLP modulated the microorganisms of the gastrointestinal tract, so that, WLG and LG had microbial composition with greater biodiversity than OG, and intestinal uniformity of the microbiota (Pielou’s evenness index) was higher in OG than WLG dogs (*P* = 0.0493) and LG (*P* = 0.0101). In addition, WLG had values of relative frequency more similar to LG than to OG.

**Conclusion:**

The fecal microbiota of the studied groups differs from each other. The weight loss program can help to reverse the changes observed in obese dogs.

## Background

Obesity is defined as the excessive accumulation of adipose tissue in the body [1] and results from a prolonged imbalance between the relative increase in energy consumption and the decrease in energy expenditure, often associated with low physical activity [2]. Several authors have described a high frequency of obesity and overweight in companion animals around the world [3–8].

The pathophysiology of obesity is complex and not fully described. In companion animals, a nutritional imbalance has been the most common cause and is mainly related to the prolonged imbalance between caloric intake and energy expenditure, which results in a chronic positive energy balance [9, 10]. Excess body weight implies numerous negative effects on health, in addition to being a risk factor for several diseases, such as orthopedic alterations [11–13], cardiovascular [14–18], respiratory [19–22] and metabolic disorders, such as insulin resistance [23] and hyperlipidemia [24, 25], immunological disorders [26] and, more recently, obesity has been attributed to causing changes in the intestinal microbiome [27].

Few studies have revealed that the host intestinal microbiota is directly associated with changes in metabolic functions, and consequently with obesity [28]. They showed that obesity affects the relative abundance of the main bacterial groups present in the intestine in mice, in addition, these animals were more efficient in obtaining energy from food than lean animals. Other studies showed an association between obesity and changes in the metabolic function of the intestinal microbiota, obese mice had a relative increase in bacterial groups that were more efficient in the metabolism of carbohydrates and lipids [29]. Another study [30] infused the gut microbiota of lean people into obese people with metabolic syndrome. They observed that after six weeks of microbiota infusion the insulin sensitivity of the receptors increased, along with the levels of butyrate-producing intestinal microbiota.

The mechanisms influenced by the intestinal microbiota with obesity are not fully elucidated, however [31] observed that the levels of serotonin, a neurotransmitter involved in the hypothalamic regulation of energy consumption, were lower in obese beagles compared to lean and gram negative bacteria, were more abundant in obese animals. It is not known whether dysbiosis induces the development of obesity or whether obesity causes dysbiosis of the gut microbiota, but obesity control involves more than energy balance.

Because of the high prevalence and health risks associated with an excess of body fat, prevention can have a positive impact on the health of pets. The appropriate time for weight control and intervention is before the gain and subsequent development of the clinical disease [32]. However, once obesity has been developed and diagnosed, a weight loss program (WLP) should be started so that the animal reestablishes the ideal BCS, thus avoiding complications related to this disease. WLP is based on three main pillars: energy restriction, physical activity, and low-calorie diet. The energy restriction for dogs is established according to the estimated ideal body weight (IBW) [32].

The ideal food for WLP has a higher concentration of protein and fiber, in addition to greater inclusion of vitamins and minerals/kcal of metabolizable energy than maintenance foods, which ensures an adequate supply of nutrients during energy restriction. The high protein content can preserve muscle mass during weight loss, fiber helps to improve satiety [33]. Some studies show that weight loss can improve quality of life and reduce the circulation of inflammatory markers in obese dogs, but few studies have evaluated changes in the fecal microbiota [26, 34–37].

Thus, the present study aimed to evaluate the effects of WLP in fecal microbiota of dogs in addition to comparing them with those of lean dogs.

## Results

### Weight loss program

The weight loss programs started on October 13, 2017 and ended with the weight loss of the last dog on July 17, 2018. The average length of weight loss of the animals included in the study was 184 ± 34.94 days and the average weekly weight loss rate was 1.14 ± 0.28%. The animals selected to compose the obese group (OG) had a higher body condition score (BCS) than the animals in the lean group (LG) (*P* < 0.001) and, after weight loss, there was no difference between the weight loss group (WLG) compared to the LG (Table 1). There were no differences in weight between the groups evaluated and, upon examination of body composition, the average amount of fat tissue was higher in the OG both in percentage (*P* < 0.001) and in kilograms (*P* = 0.0053) although, after weight loss, these values were similar to the lean group. In addition, the weight loss program (WLP) resulted in an increase in lean mass (%) of obese animals (*P* < 0.001).

**Table 1:** Body weight, BCS and body composition of the obese (OG), lean (LG) and weight loss group (WLG)

Variable	OG	LG	WLG	SEM	*P *value
BCS^1^	9^A^	5^B^	5.7^B^	0.8819	< 0.0001
Body weight (kg)	22.8	14.96	17.67	3.309	0.2529
Fat mass (kg)	8.42^A^	2.89^B^	4.34^B^	1.5978	0.0053
Lean mass (kg)	14.37	12.29	13.33	2.3602	0.8259
Fat mass (%)	36.93^A^	17.21^B^	24.05^B^	2.0391	< .0001
Lean mass (%)	63.06^A^	82.48^B^	75.95^B^	2.0391	< 0.0001

^1^Body condition score

^A^^−^^B^Means followed by different letters in the lines differ by 5% in the Tukey test adjusted by Proc Mixed

### Microbiota diversity

The results obtained from the T-RFLP technique (diversity and richness), 30 fecal samples (10 samples of OG, 10 samples of WLG and 10 samples of LG) were chosen for the characterization of the bacterial community, which were sequenced in “multiplexing” on the MiSeq Illumina platform. In this study 1,384,661 sequences were generated and these were grouped into OTUs using the UPARSE-OTU algorithm and 97% of similarity. The average of OTUs and sequences generated per sample was 64.85 ± 19.41 and 46.15 ± 8.39 respectively.

**Fig. 1:**
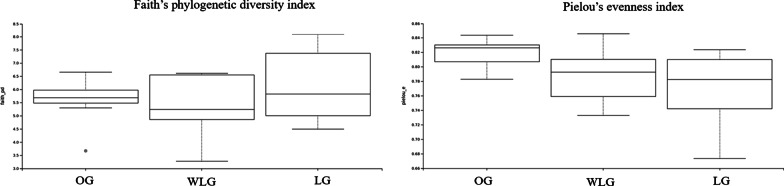
Faith phylogenetic diversity index and Pielou´s evenness index of the experimental groups (OG: obese group; WLG: weight loss group; LG: lean group)

**Fig. 2:**
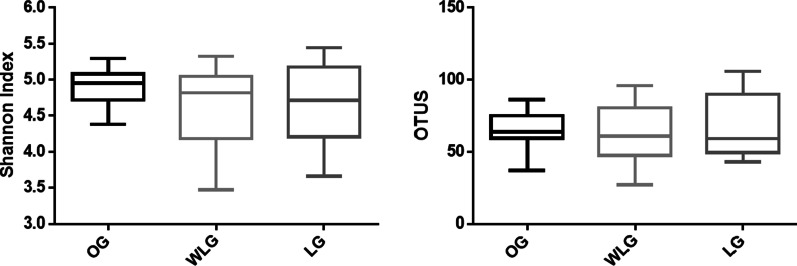
Shannon alpha-diversity index and OTUs count of experimental groups (OG: obese group; WLG: weight loss group; LG: lean group)

From the alpha diversity indexes evaluated in this study, no differences were found in the richness of microorganisms through the Faith’s phylogenetic diversity index, Shannon alpha-diversity index and OTUs count among OG, WLG and LG (*P* > 0.05). However, Pielou's evenness index was higher in OG than in WLG (*P* = 0.0493) and LG (*P* = 0.0101) dogs and no differences between WLG and LG (*P* = 0.6501) (Figs. 1 and 2).

**Fig. 3:**
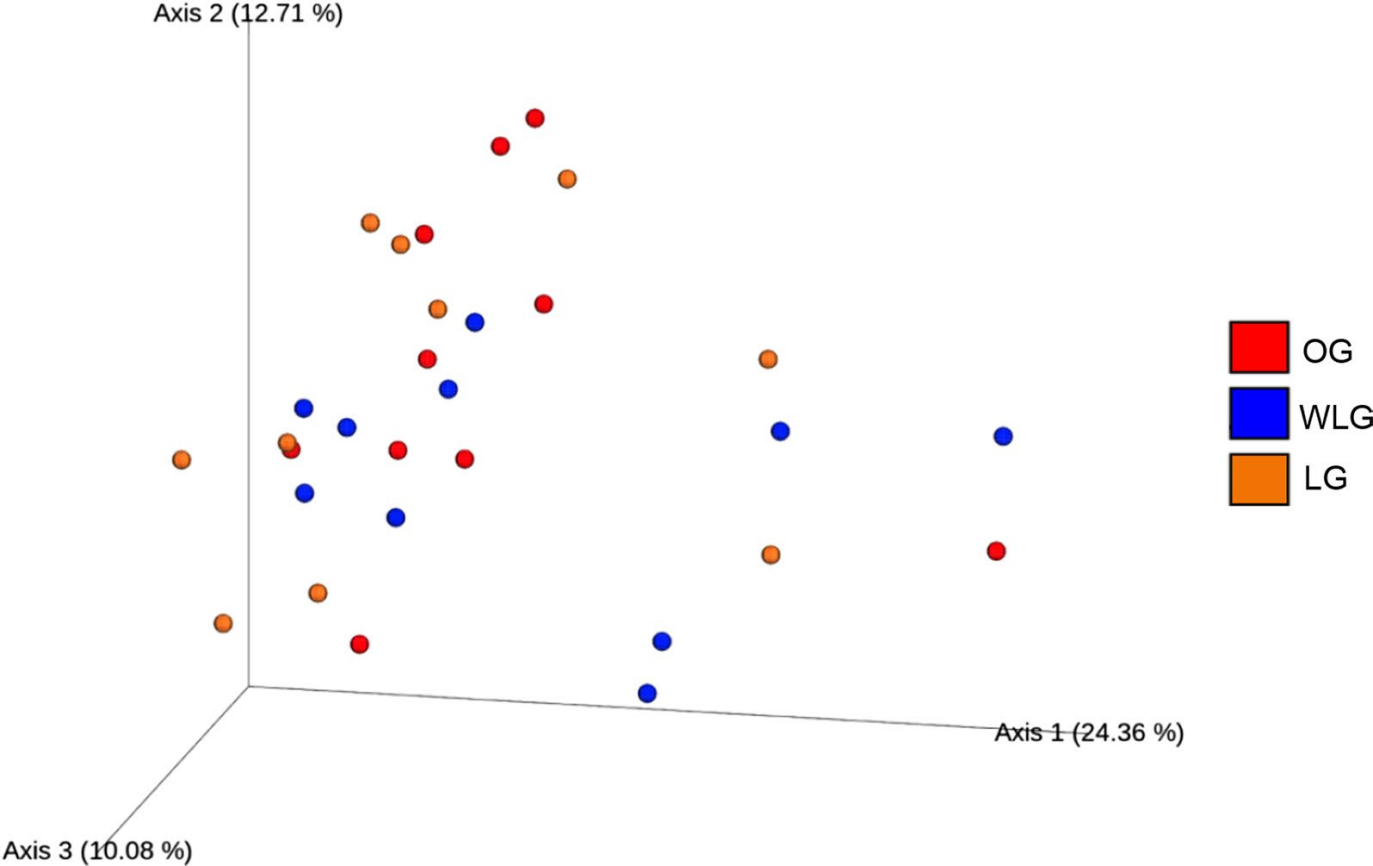
Three-dimensional analysis of principal components with the absolute data obtained by sequencing of the samples of the experimental groups (OG: obese group; WLG: weight loss group; LG: lean group)

Beta diversity was assessed by principal component analysis (PCA) (Fig. 3), the graphic shows a tendency to group experimental treatments on the same axis. Analysis was confirmed by PERMANOVA and no differences in the microbiome between the groups (*P* > 0.05) were found.

### Comparison between bacterial groups

Bacteria taxa were compared when the referring taxa was represented in all groups so that, 5 phyla, 17 families, 27 genera and 33 species had valid values for statistical analysis.

**Fig. 4:**
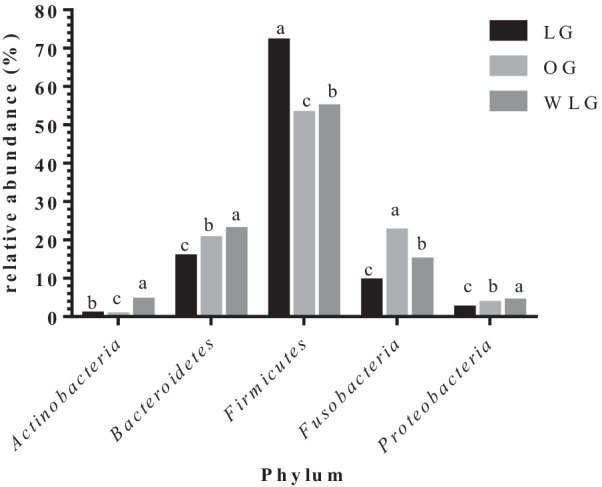
Relative abundance of each phyla found in experimental groups (OG: obese group; WLG: weight loss group; LG: lean group). ^a,b,c^Means followed by different letters differ by 5% in the Tukey test adjusted by PROC GLIMMIX

**Fig. 5:**
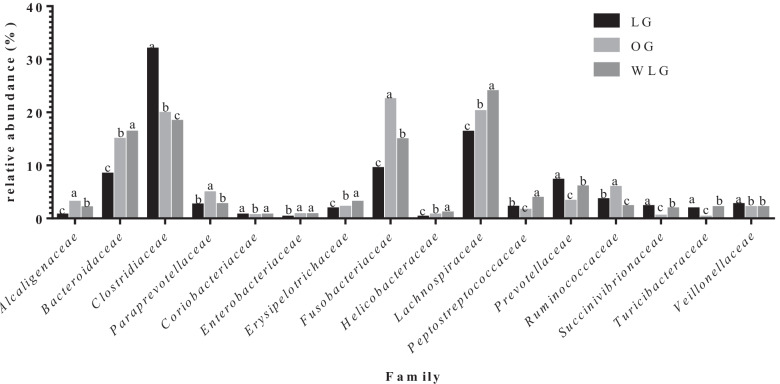
Relative abundance of families found in experimental groups (OG: obese group; WLG: weight loss group; LG: lean group). ^a,b,c^Means followed by different letters differ by 5% in the Tukey test adjusted by PROC GLIMMIX

**Fig. 6:**
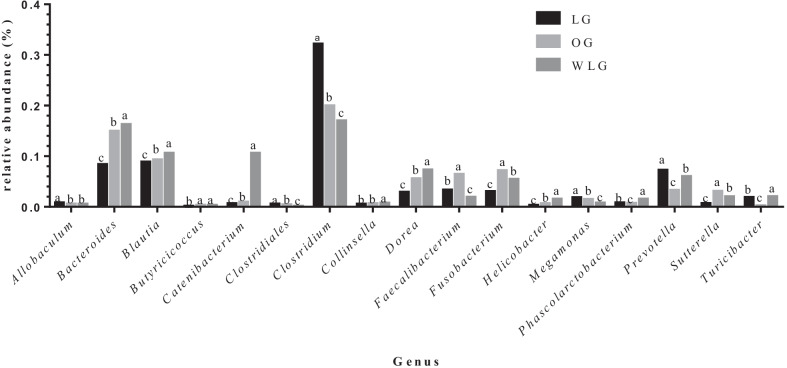
Relative abundance of genera found in experimental groups (OG: obese group; WLG: weight loss group; LG: lean group). ^a,b,c^Means followed by different letters differ by 5% in the Tukey test adjusted by PROC GLIMMIX

Regarding phylum, family and genera respectively, *Firmicutes* (71%), *Clostridiaceae* (31%) and *Clostridium* (0.32%) had the highest relative abundances. On the other hand, *Actinobacteria* (0.5%), *Clostridiales* (0.12%) and *Butyricicoccus* (0.001%) had the lowest relative abundances. Results for each bacteria taxa were described in Figs. 4, 5 and 6.

The WLG presented intermediate values for relative frequency (between OG and LG) for the phyla: *Actinobacteria*, *Firmicutes* and *Fusobacteria* (*P* < 0.0001); families: *Alcaligenaceae, Coriobacteriaceae, Fusobacteriaceae, Lachnospiraceae, Peptostreptococcaceae, Prevotellaceae, Ruminococcaceae, Succinivibrionaceae, Turicibacteriaceae* and *Paraprevotellaceae* (*p* < 0.0001) and genera: *Faecalibacterium*, *Fusobacterium*, *Phascolarctobacterium*, *Prevotella*, *Sutterella, Turicibacter* and *Eubacterium* (*P* < 0.0001). The other differences found between the groups in the different taxa are described in Tables 2, 3, and 4.

**Table 2:** Relative abundance of phyla found in experimental groups

Phylum	Treatments			*P* value
	LG	OG	AWL	
	Mean ± sem	Mean ± SEM	Mean ± SEM	
*Actinobacteria*	0.7 ± 0.01^B^	0.5 ± 0.01^C^	4.3 ± 0.04^A^	< .0001
*Bacteroidetes*	15.6 ± 0.05^C^	20.4 ± 0.06^B^	22.8 ± 0.06^A^	< .0001
*Firmicutes*	71.9 ± 0.07^A^	53.0 ± 0.07^C^	54.8 ± 0.07^B^	< .0001
*Fusobacteria*	9.4 ± 0.04^C^	22.4 ± 0.06^A^	14.8 ± 0.05^B^	< .0001
*Proteobacteria*	2.3 ± 0.02^C^	3.5 ± 0.03^B^	4.1 ± 0.03^A^	< .0001

OG: obese group; WLG: weight loss group; LG: lean group

^A,B,C^ Means followed by different letters differ by 5% in the Tukey test adjusted by PROC GLIMMIX

**Table 3:** Relative abundance of family found in experimental groups

Family	Treatments			*P* value
	LG	OG	AWL	
	Mean ± SEM	Mean ± SEM	Mean ± SEM	
*Lachnospiraceae*	0.6 ± 0.01^C^	3.0 ± 0.03^A^	1.97 ± 0.02^B^	< .0001
*Bacteroidaceae*	8.3 ± 0.04^C^	14.9 ± 0.05^B^	16.25 ± 0.05^A^	< .0001
*Clostridiaceae*	31.9 ± 0.07^A^	19.8 ± 0.06^B^	18.27 ± 0.05^C^	< .0001
*Coriobacteriaceae*	0.6 ± 0.02^A^	0.5 ± 0.01^B^	0.6 ± 0.01^A^	0.0005
*Enterobacteriaceae*	0.2 ± 0.01^C^	0.7 ± 0.02^B^	2.3 ± 0.02^A^	< .0001
*Erysipelotrichaceae*	1.8 ± 0.02^C^	2.1 ± 0.02^B^	3.0 ± 0.03^A^	< .0001
*Fusobacteriaceae*	9.4 ± 0.04^C^	22.4 ± 0.06^A^	14.8 ± 0.05^B^	< .0001
*Helicobacteraceae*	0.2 ± 0.01^C^	0.6 ± 0.01^B^	1.0 ± 0.02^A^	< .0001
*Lachnospiraceae*	16.2 ± 0.05^C^	20.1 ± 0.06^B^	23.9 ± 0.06^A^	< .0001
*Peptostreptococcaceae*	2.1 ± 0.02^B^	1.5 ± 0.02^C^	3.8 ± 0.03^A^	< .0001
*Prevotellaceae*	7.2 ± 0.05^A^	3.2 ± 0.04^C^	5.9 ± 0.04^B^	< .0001
*Ruminococcaceae*	3.5 ± 0.03^B^	5.8 ± 0.03^A^	2.2 ± 0.02^C^	< .0001
*Succinivibrionaceae*	2.2 ± 0.03^A^	0.4 ± 0.02^C^	1.8 ± 0.02^B^	< .0001
*Turicibacteraceae*	1.8 ± 0.02^B^	0.2 ± 0.01^C^	2.0 ± 0.02^A^	< .0001
*Veillonellaceae*	2.6 ± 0.02^A^	2.0 ± 0.02^B^	2.0 ± 0.02^B^	< .0001
*[Paraprevotellaceae]*	2.5 ± 0.03^B^	4.8 ± 0.04^A^	2.6 ± 0.03^B^	< .0001

OG: obese group; WLG: weight loss group; LG: lean group

^A,B,C^Means followed by different letters differ by 5% in the Tukey test adjusted by PROC GLIMMIX

**Table 4:** Relative abundance of genus found in experimental groups

Genus	Treatments			*P* value
	LG	OG	AWL	
	Mean ± SEM	Mean ± SEM	Mean ± SEM	
*Allobaculum*	0.0079 ± 0.0001^A^	0.0055 ± 0.0001^B^	0.0052 ± 0.0001^B^	< .0001
*Bacteroides*	0.0834 ± 0.0004^C^	0.1490 ± 0.0005^B^	0.1625 ± 0.0005^A^	< .0001
*Blautia*	0.0884 ± 0.0004^C^	0.0928 ± 0.0004^B^	0.1057 ± 0.0005^A^	< .0001
*Butyricicoccus*	0.0012 ± 0.0001^B^	0.0030 ± 0.0001^A^	0.0028 ± 0.0001^A^	< .0001
*Catenibacterium*	0.0061 ± 0.0001^C^	0.0092 ± 0.0002^B^	0.0605 ± 0.0007^A^	< .0001
*Clostridiales*	0.0056 ± 0.0001^A^	0.0041 ± 0.0001^B^	0.0012 ± 0.0001^C^	< .0001
*Clostridium*	0.3215 ± 0.0007^A^	0.1995 ± 0.0006^B^	0.1697 ± 0.0006^C^	< .0001
*Collinsella*	0.0053 ± 0.0001^B^	0.0060 ± 0.0001^B^	0.0073 ± 0.0002^A^	< .0001
*Dorea*	0.0287 ± 0.0002^C^	0.0556 ± 0.0003^B^	0.0729 ± 0.0004^A^	< .0001
*Enterobacteriaceae*	0.0026 ± 0.0001^C^	0.0073 ± 0.0002^B^	0.0234 ± 0.0004^A^	< .0001
*Erysipelotrichaceae*	0.0035 ± 0.0001^A^	0.0027 ± 0.0001^B^	0.0010 ± 0.0001^C^	< .0001
*Faecalibacterium*	0.0333 ± 0.0003^B^	0.0643 ± 0.0004^A^	0.0188 ± 0.0002^C^	< .0001
*Fusobacteriaceae*	0.0671 ± 0.0004^C^	0.1595 ± 0.0005^A^	0.0987 ± 0.0004^B^	< .0001
*Fusobacterium*	0.0303 ± 0.0003^C^	0.0716 ± 0.0004^A^	0.0542 ± 0.0003^B^	< .0001
*Helicobacter*	0.0028 ± 0.0001^C^	0.0063 ± 0.0001^B^	0.0108 ± 0.0002^A^	< .0001
*Lachnospiraceae*	0.0175 ± 0.0002^B^	0.0244 ± 0.0002^A^	0.0126 ± 0.0002^C^	< .0001
*Megamonas*	0.0181 ± 0.0002^A^	0.0145 ± 0.0002^B^	0.0076 ± 0.0001^C^	< .0001
*Peptostreptococcaceae*	0.0216 ± 0.0002^B^	0.0148 ± 0.0002^C^	0.0369 ± 0.0003^A^	< .0001
*Phascolarctobacterium*	0.0077 ± 0.0001^B^	0.0068 ± 0.0001^C^	0.0150 ± 0.0002^A^	< .0001
*Prevotella*	0.0723 ± 0.0005^A^	0.0325 ± 0.0004^C^	0.0599 ± 0.0004^B^	< .0001
*Ruminococcaceae*	0.0037 ± 0.0001^AB^	0.0042 ± 0.0001^A^	0.0031 ± 0.0001^B^	< .0001
*Sutterella*	0.0066 ± 0.0001^C^	0.0304 ± 0.0003^A^	0.0197 ± 0.0002^B^	< .0001
*Turicibacter*	0.0186 ± 0.0002^B^	0.0022 ± 0.0001^C^	0.0202 ± 0.0002^A^	< .0001
*[Eubacterium]*	0.0027 ± 0.0001^B^	0.0069 ± 0.0001^A^	0.0026 ± 0.0001^B^	< .0001
*[Paraprevotellaceae]*	0.0143 ± 0.0003^B^	0.0175 ± 0.0003^A^	0.0166 ± 0.0003^A^	0.0006
*[Prevotella]*	0.0202 ± 0.0002^B^	0.0395 ± 0.0003^A^	0.0205 ± 0.0003^B^	< .0001
*[Ruminococcus]*	0.0257 ± 0.0002^B^	0.060 ± 0.0002^B^	0.0463 ± 0.0003^A^	< .0001

*OG* obese group, *WLG* weight loss group, *LG* lean group

^A,B,C^Means followed by different letters differ by 5% in the Tukey test adjusted by PROC GLIMMIX

## Discussion

The percentage of average body weight loss per week was within the recommended range for dogs (1 to 2%) [38]. These results are of paramount importance, as they demonstrate that the weight loss program in all animals was carried out in a healthy and appropriate manner.

All animals in the OG had BCS (9/9) [39] and, when examining body composition, the average percentage of fat was greater than 40%. The key point of a weight loss program is the reduction of energy intake [40, 41], in order to promote the negative energy balance associated with maintaining lean mass [42], and this was observed in our study, because despite the difference in fat mass composition, lean mass did not change after weight loss, except when expressed as a percentage. This is essential, as the body's muscle tissue is metabolically active and guarantees greater energy expenditure [38].

In this study, no differences were observed in beta diversity, as well as in the alpha diversity and Faith's phylogenetic diversity and Shannon alpha-diversity indexes as well as the simple OTUs count. However, Pielou's evenness index showed greater uniformity among the microbial species were found in the OG samples, when compared to the WLG and LG groups. This result may suggest that weight loss increased the biodiversity of emaciated dogs.

According to [43], an important factor related to dysbiosis may be the loss of total microbial diversity. Recently, a study with dogs [44], concluded that increased diversity may be an important factor or even a marker of a healthy canine microbiota, however, further studies are needed to confirm this hypothesis in dogs. Another study [27] evaluated beagle dogs both in ideal body condition and obese. They also did not observe differences in the Shannon diversity index (alpha diversity) and the number of operational taxonomic units (OTU) between groups. And another study finally [45] found a lower number of OTUs for genera and species in dogs after weight loss, however, the estimated total number of species was higher in these same animals, despite the maintenance of uniformity and diversity of species. The work concluded that the biodiversity of the microbiota increased between the beginning of weight loss and the end.

In this study, it is possible to observe that some of the relative frequency of bacterial groups in WLG is intermediate to LG than to OG (Fig. 4). Studies in humans, rats and even dogs have shown that weight loss causes changes in the intestinal microbiota, and some bacterial groups can return to proportions similar to those of lean animals. An example is the relative abundance of *Firmicutes* phyla, which is found increased in obese individuals, while *Bacteroidetes* is decreased, but after weight loss individuals tend to have a microbiota more similar to lean individuals, with an increased relative abundance of *Bacteroidetes* phyla and decreased *Firmicutes* [27, 46–49].

In our study, the relative abundance of the phylum *Bacteroidetes* increased after weight loss, corroborating the above-mentioned studies. On the other hand, *Firmicutes* phylum was found in greater proportion in WLG and LG than in OG. According to [27] *Firmicutes* is the most abundant phylum in dogs despite of BCS and a previous study [31] indicated that *Proteobacteria* was the predominant phylum in obese dogs and *Firmicutes* in lean dogs. Ley et al.[46] were the first to report a 50% reduction in the abundance of the phylum *Bacteroidetes* and a proportional increase in *Firmicutes* in obese rats. Shortly afterwards they found similar results in humans [29]. In contrast, other studies have observed the opposite [50] or no differences in the proportions of *Bacteroidetes* and *Firmicutes* between thin and obese individuals [51, 52]. Sanchez et al. [37] reported that the increase in *Bacteroidetes* may have occurred due to the increase in the *Bacteroides* and *Paraprevotella* genera after weight loss, similar results were observed with dogs in our study, which showed an increase in the genus *Bacteroides* and an increase in an unconfirmed genus of the *Paraprevotellaceae* family. As for a study by Park et al. [31], OG had a greater abundance of *Proteobacteria* and *Fusobacteria* phyla when compared to LG, and dogs after weight loss, and decreased the population of *Proteobacteria*. The *Actinobacteria* phylum also showed an increase in dogs after weight loss, a similar result was reported in cats [53], and they associated this increase to the *Collinsella* genera, which also occurred in this study.

Within the *Firmicutes* phylum, the families *Erysipelotrichaceae*, *Turicibacteraceae*, *Veillonellaceae*, *Peptostreptococcaceae*, *Clostridiaceae*, *Lachnospiraceae* and *Ruminococcaceae* were found in all groups. *Lachnospiraceae* and *Ruminococcaceae* had greater abundances in the OG when compared to the other two groups, this may have occurred due to the higher concentration of *Butyricicoccus* and *Faecalibacterium* genera in obese animals. In a study carried out with rats, a high protein—low carbohydrate diet led to a decrease in the *Lachnospiraceae* and *Ruminococcaceae* families, suggesting that they may be important competitors of *C. difficile* for amino acids in the lumen. They further discuss that the high protein—low carbohydrate diet would lead to an abundance of oligopeptides and free amino acids in the lumen and could provide a selective advantage that leads to *C. difficile* overgrowth when associated with loss of *Lachnospiraceae* and *Ruminococcaceae* [54]. Furthermore, *Lachnospiraceae*, in particular, is a dominant bacterial family in the intestinal microbial communities of many mammals. [54]. In contrast, the genera *Dorea* and *Ruminococcus*, belonging to the families mentioned above, were found in higher concentrations in animals after weight loss. Previous studies have shown a decrease in the *Dorea* genus after weight loss [37, 45], on the other hand, the increase in *Ruminococcus* can be explained by the use of the diet, which has high protein, as previously observed by [37, 55] in dogs.

However, in this same phylum, the families *Veillonellaceae* and *Clostridiaceae* had greater abundance in the LG, the genera *Meganomas* belonging to the *Veillonellaceae* family presented greater abundance in LG and smaller in WLG, which may be related to the weight loss diet, which has already been previously reported in other studies [27, 37]. According to Pilla et al. [56], the relative abundance of the *Clostridiaceae* family is positively correlated with dietary protein digestibility and negatively with fecal protein content, the diet used for weight loss in this study contained approximately 10% of dietary fiber, which may lead to decreased protein digestibility.

In the WLG the predominant families were *Erysipelotrichaceae*, *Turicibacteraceae*, *Peptostreptococcaceae* and *Lachnospiraceae*. According to Bermingham et al. [57] *Erysipelotrichaceae* were positively correlated with a number of markers associated with carbohydrate digestion, including diets with high fiber content and short chain fatty acids production. The same authors observed a negative correlation between *Erysipelotrichaceae* and markers of protein metabolism in the intestinal tract. Among members of the *Erysipelotrichaceae* family, we found an effect on the genus *Allobaculum*, *Catenibacterium*, and *Turicibacter* which were identified as part of a healthy microbiota in dogs [56, 57]. The increase of *Turicibacteraceae* in the WLG may be due to the presence of FOS in the diet composition. In other studies [56, 58], inulin-type fructans prebiotics had also increased *Firmicutes* but from families *Erysipelotrichaceae* and *Turicibacteraceae*. Although *Peptostreptococcaceae* showed greater abundance in the WLG, the *Clostridium* genera, belonging to this family, was underrepresented. In an experiment with healthy dogs, the presence of high fiber and prebiotics led to a relative increase in this family [59]. Other studies have shown that after weight loss, dogs had a decrease in genus *Clostridium* [45] and the greater abundance of the *Clostridia* class was associated with an obese phenotype in humans, and it is reported to decrease after weight loss [60]. In addition to the increase in *Lachnospiraceae*, the WLG had an increase in *Blautia* genera, as well as other families of the phylum *Firmicutes*, dogs that consume high protein diets show an increase in *Lachnospiraceae* [55].

Within the phylum *Bacteroidetes*, the population of *Bacteriodaceae* family increased after weight loss, followed by OG and LG, as well as the genus *Bacteroides* belonging to this family. In a study carried out by Bermingham et al. [57], they showed a positive correlation between the presence of *Bacteroidaceae* and the dietary fiber content. Just as in our study *Bacteriodes* was the only genus found within *Bacteriodaceae* [57]. Relative count of *Prevotellaceae* family, was greater in LG followed by WLG and smaller in OG, as well as the genus *Prevotella*. According to Park et al. [61] *Prevotella* is one of the two most abundant genera in healthy dogs, so it is common to be found on LG in higher relative counts.

The phylum *Actinobacteria*, family *Coriobacteriaceae* and the genus *Collinsella* showed lower abundance in OG when compared to WLG. In the study carried out by Pallotto et al. [53], they compared fecal microbiota of obese cats and after a weight loss program and observed an increase in *Actinobacteria* with weight loss which was primarily attributable to an increase in *Bifidobacterium spp* and *Collinsella spp*.

*Fusobacteria* phylum showed a decrease in the *Fusobacteriaceae* family after weight loss, as did the *Fusobacterium* genus. Diverging results were found by Sanchez et al.[37] who observed an increase in this bacterial group in dogs after weight loss. However, in this study when compared to the lean group, we observed that the WLG presented intermediate values between the OG and LG, thus showing that there was an approximation of the standard microbiota.

Finally, in the *Proteobacteria* phylum, the *Enterobacteriaceae* and *Helicobacteraceae* families increased their proportion after weight loss, as did the genus *Helicobacter*. While *Succinivibrionaceae* followed by greater abundance in LG, by WLG and smaller in OG. No previously published studies have shown differences between dogs after weight loss for these families and genera. Meanwhile *Succinivibrio* and *Anaerobiospirillum* are both succinate-producing members of the *Succinivibrionaceae* family of the *Gammaproteobacteria*, and are recognized as part of the normal fecal microbiota of dogs and cats [62].

### Particularities and limitations

The fecal microbiota can be influenced by several factors, such as sequencing method, sample type, genotype, age, sex, environment and diet [63, 64]. Therefore, in this study, in addition to taking care of animal standardization, collection and sequencing methods, the design was carried out to had special attention in two moments: the period of diet acclimation and the weight loss program. The diet’s acclimation period, in which obese and lean dogs were included at the beginning of the study, aimed to reduce the variation in composition between the different diets consumed by the animals before participating in the study, a factor cited as important in the characterization of the fecal microbiota [27, 64]. Thus, the results of the microorganism profile observed among obese and control animals had less dietary influence, despite of living in different households.

This study is one of the few that evaluated the effect of weight loss on the fecal microbiota of naturally obese dogs [37]. It provided a detailed experimental comparison of phyla, family, genera and bacterial species found in the feces of dogs in the obese condition and after the reduction of 20% of the body weight of the same animals and of dogs in an ideal BCS.

A fact that can be considered as a limitation is the variability between control and low-calorie diets. However, in this study, the aim was to evaluate the influence of WLP (high-protein-high-fiber diet and energy restriction) on the fecal microbiota of obese dogs and after loss of 20% of the initial weight of the study. In addition, another study [37] had evaluated the effect of a commercial diet for weight loss, with a similar profile to that used in this study, on the fecal microbiota of obese dogs before and after period of consumption of a weight loss diet. They also evaluated animals that did not complete the WLP, and these dogs showed no significant differences in their fecal microbiota before and after starting the WLP. Similar results were shown in the study by Kieler et al. [65], which evaluated the fecal microbiota of overweight dogs after a WLP. Still on this study, the dogs were followed for 12 weeks, and it is not clear how many dogs reached an ideal body weight. The main finding was the decrease in abundance of the *Megamonas* genera, which correlated with a higher rate of weight loss during the 12-week weight loss of the WLP [65]. In our study, we also observed a decrease in the *Megamonas* genera (Fig. 4) after weight loss, which can be attributed to of the use of commercial diets for weight loss. However, the role of *Megamonas* in obesity is unclear and deserves further investigation.

## Conclusions

The results found, under the conditions of the present study, confirm the hypothesis that the composition of the fecal microbiota of obese dogs is different from the microbiota of dogs in an ideal body condition score. In addition, the weight loss program consisting of energy restriction associated with the use of a low-calorie commercial food (low energy and high fiber and protein) promoted changes in the fecal microbiota. In addition, WLG presented values of relative frequency more similar to LG than to OG.

## Methods

### Animals, location and standardization of diet

This study included 20 dogs that were screened and monitored at the Pet Nutrology Research Center (CEPEN Pet) located at School of Veterinary Medicine and Animal Science (FMVZ) at USP in the city of Pirassununga-SP. In the OG were included 10 female dogs with different breeds, sterilized, aged between 1 and 9 years (6.29 ± 1.804), body condition score (BCS) 9/9 according to Laflamme et al. [39], muscle mass score (MMS) 2 or 3 according to Michel et al. (2011) [66] and with fat mass greater than 30% determined by the deuterium isotope dilution method [23, 67]. The LG was composed by 10 healthy female dogs, aged between 1 and 4 years (2.09 ± 0.842), with ideal BCS (5/9), MMS 2 or 3, and maximum body fat of 15% [23, 39, 66, 67].

All dogs underwent a complete physical examination, nutritional anamnesis, physical exams including BCS and MMS evaluation, complete blood count and biochemical profile tests [albumin, glucose, total protein, urea, creatinine, alkaline phosphatase, cholesterol, triglycerides, aspartate aminotransferase (AST) and alanine aminotransferase (ALT)] in order to exclude animals with concomitant illnesses.

After completing the screening step, the animals of OG and LG started to receive a maintenance diet (Golden Formula—Adult Dogs/Chicken and Rice, Premier Pet Indústria e Comércio Ltda, Dourado, Brazil) to standardize the diet. They received this diet for 28 days, before the beggining of the study. The chemical composition (organic matter) and the ingredients list is shown in Table 5.

**Table 5:** Chemical composition (organic matter) and list of ingredients^1^ of the control food used in the study

Item	%	Unit/kg	Unit/1000 kcal ME^2^
Moisture	7.96	79.60 g	26.70 g
Crude protein	25.51	255.10 g	85.60 g
Fat	12.60	126.00 g	42.30 g
Ash	5.30	53.00 g	17.80 g
Dietary fiber	1.91	19.10 g	6.40 g
Calcium	1.13	11.30 g	3.70 g
Phosphorus	0.85	8.50 g	2.80 g
Metabolizable energy	–	3795 kcal	–

^1^Meat meal, chicken viscera meal, isolated pork protein, ground whole corn, rice chops, beet pulp, defatted rice bran, chicken fat, pork fat, flaxseed, pork and chicken hydrolyzate, propionic acid, antioxidants BHA and BHT, potassium chloride, sodium chloride, dry brewer's yeast, yeast cell wall, vitamin A, vitamin B12, vitamin C, vitamin D3, vitamin E, vitamin K3, folic acid, pantothenic acid, biotin, choline chloride, niacin, pyridoxine, riboflavin, thiamine, potassium iodide, selenium proteinate, copper sulphate, iron sulphate, manganese sulphate, zinc sulphate

^2^Metabolizable energy

The maintenance energy requirement (MER) of each animal was calculated in order to achieve 95 kcal × (body weight)^0.75^ kcal/day [68]. The daily amount of food provided was determined by dividing MER by metabolizable energy of the diet used in the study.

After adapting the control diet, the first fecal collection was performed to analyze the microbiota in the OG and CG. Then, dogs belonging to the GO were included in the WLP, when they reached a 20% reduction in body weight, they became WLG.

### Body composition

The body composition of the animals was determined by the deuterium isotope dilution method. After an 8-h fasting period, deuterium oxide (10%) solution was administered subcutaneously (1 mL/kg) of body weight of a 10%. Blood samples (3 mL) were collected by jugular vein puncture immediately before and 2 h after deuterium oxide inoculation. Samples were processed for serum extraction and later stored at -20 °C until analysis.

Deuterium enrichment of the samples was determined by isotope ratio mass spectrometry (IRMS, Calixto System—Sercon Ltd, Gateway, United Kingdom) at the Isotope Ratio Mass Spectrometry Laboratory of the Department of Internal Medicine, Faculty of Medicine of Ribeirão Preto—USP, Ribeirão Preto, Brazil. The dog’s body composition were determined according to the methodology described by [67] and adapted for dogs by [23].

After quantifying body water, total lean mass and fat mass by difference (expressed in percentage) were calculated. This assessment was performed after the animals' acclimation period in the OG and LG to quantify the percentage of fat mass of the individuals included in the study. After the weight loss program, the WLG animals were also submitted to the same evaluation.

### Weight loss program

The WLP was carried out following the recommendations by [32], therefore, the energy requirement for weight loss (ERWL) of animals was estimated to provide 70 kcal × (ideal body weight)^0.75^ per day.

The ideal body weight was calculated by subtracting 20% [22, 23, 32] of the initial body weight and the daily amount of food provided was determined by division of ERWL by the metabolizable energy of the low-calorie diet used in the study (Premier Clinical Nutrition Obesity – Medium and Large Dogs, Premier Pet Indústria e Comércio Ltda, Dourado, Brazil). The chemical composition (organic matter) and the ingredients list of this diet are shown in Table 6.

**Table 6:** Chemical composition (organic matter) and ingredients list^1^ of the hypocaloric food used in the study

Item	%	Unit/kg	Unit/1000 kcal de ME^2^ (g)
Moisture	8.14	81.40 g	27.30
Crude protein	36.92	369.20 g	123.90
Fat	10.27	102.70 g	34.50
Ash	5.61	56.10 g	18.80
Dietary fiber	10.37	103.70 g	34.80
Calcium	0.98	9.80 g	3.30
Phosphorus	0.79	7.90 g	2.60
Metabolizable energy	–	2979 kcal	–

^1^Poultry meal, wheat gluten, isolated pig protein, powdered pig plasma, dehydrated egg, pea flour, barley, rice cooker, cellulose, beet pulp, chicken fat, fish oil, pig hydrolyzate and chicken, propionic acid, antioxidant BHA, β-glucan, potassium chloride, sodium chloride, yucca extract, fructooligosaccharides, hydrolyzed gelatin (2.5%), L-carnitine, dry brewer's yeast, yeast cell wall (source of MOS), taurine, vitamin A, vitamin B12, vitamin C, vitamin D3, vitamin E, vitamin K3, folic acid, pantothenic acid, biotin, choline chloride, niacin, pyridoxine, riboflavin, thiamine, iron chelate amino acid, iodide potassium, manganese amino acid chelate, selenium proteinate, copper sulfate, iron sulfate, zinc sulfate, manganese sulfate, zinc chelate amino acid, copper chelate amino acid

^2^Metabolizable energy

The amount of food was offered to the animals by the owners, two to three times a day. They received a measuring cup with an indication of the prescribed amount to be offered. The low-calorie diet was given for all obese dogs. The animals were reassessed every 15 days to verify: BCS, MMS, BW, calculations of the weekly weight loss rate and, when necessary, adjustments in the amount of food. In addition, owners were encouraged to take their dogs to practice physical activity (a walk around 3 times a week, for at least 15 min). When the animals achieved a 20% reduction in their body weight, they were discharged from the WLP, and became part of the WLG.

### Microbiota analysis

The fecal collection from the CG and OG occurred after 28 days of acclimation to the control diet, in order to standardize the diet of the 20 animals in the study. The collection of feces from the WLG was carried out after the end of the WLP.

Owners received a brief training about how to collect feces on sterile gloves and then to store in an sterile cup. All owners collected feces in their own houses. In addition, owners were instructed to collect the feces at the exact moment their dogs were defecating, that is, without contact with any contaminated surface, and then store the sample in a freezer at −20 °C. Immediately after collecting the material, all participants were asked to contact the veterinarians conducting this study (H.T.M and T.H.A.V) so that feces were taken to store it in a freezer at -80 °C until the analysis was performed.

The samples were sent to the BPI laboratory, located in Botucatu, São Paulo, Brazil and the total DNA of each sample was extracted using the ZR Fungal/Bacterial DNA MiniPrep™ kit (Zymo Research code D6005) according to the manufacturer's protocol.

The 16S rRNA gene amplification reactions were performed in triplicate samples, and a final volume of 20μL was obtained, containing 10μL of GoTaq® Colorless Master Mix 2× (Promega, USL), 0.3 μM of forward oligonucleotide and 0.3 μM of reverse oligonucleotide, 1μL of genomic DNA and sufficient sterile ultrapure water to make up to 20 μL. The amplification program consisted of initial denaturation at 95ºC for 5 min., followed by 29 cycles of denaturation at 95ºC for 30 s., annealing at 55ºC for 1 min; extension at 72ºC for 1 min. 30 s. and a final extension at 72 °C for 10 min. Amplification reactions were conducted in a Veriti™ Thermal Cycler (Applied Biosystems) thermocycler. For amplification, the universal forward primer used were (5'-ATGATACGGCGACCACCGAGATCTACAC TATGGTAATT GT GTGCCAGCMGCCGCGGTAA-3'). After PCR reaction, the amplification of each sample was confirmed by electrophoresis in 2% agarose gel stained with Gel Red (Uniscience). ~ 300 bp (amplicon size).

The PCRs were submitted to purification steps using Agencourt AMPure XP magnetic bead (Beckman Coulter), to remove very small fragments from the total population of molecules and primers and reaction. After this step, quantification was carried out using the real-time PCR methodology in a QuantStudio 3 Real Time thermocycler (Applied Biosystems) and KAPA-KK4824 Kit (Library Quantification Kit—Illumina/Universal), all according to the manufacturer's protocol.

An equimolar pool of DNA was generated by normalizing all samples to 3 nM for sequencing, which was conducted using the next-generation Illumina MiSeq sequencing system (Illumina® Sequencing) and MiSeq Reagent Kit kit V2 Micro 300 cycles—2 × 150 bp reading.

Analyzes were performed using the QIIME2 platform ver 2019.10. The sequences were quality filtered and grouped into taxonomic units (OTUs) using 97% similarity between the sequences. A representative sequence of each OTU was used to construct a phylogenetic tree that was used in the beta-diversity analyses. The proportion of each OTU in each sample was used for alpha and beta-diversity analyses. The alpha-diversity indices were calculated: Shannon's diversity index (quantitative index that measures the richness of each sample); amount of OTUs in each sample and Evenness (or Pielou's Evenness; a measure of the uniformity of each sample). The sequences were also compared to a database (Green Genes, 13.5) for taxonomic analysis.

### Calculations and statistical analysis

The data with normal distribution were subjected to analysis of variance at 5% significance, using the PROC MIXED of the Statistical Analysis System program, version 9.3 (SAS, 1995) and, when differences between the means were detected, these were compared by the Tukey test. For the characterization of the microbiota, the abundances observed for each Phylum and Gender were evaluated by the Generalized Linear Model, considering binomial distribution and using the logit link function. The model included fixed effects of Groups (Control, Obese and Weight Loss), in addition to the random effects of animal and residue. All analyzes were performed using the PROC GLIMMIX procedure, using the Statistical Analysis System program, version 9.3 (SAS Institute Inc., Cary, NC, USA). Values of *p* < 0.05 were considered significant.

## Data Availability

The dataset supporting the conclusions of this article is included within the article.

## Abbreviations

ALT: Alanine aminotransferase
AST: Aspartate aminotransferase
BCS: Body condition score
BW: Body weight
ERWL: Energy requirement for weight loss
IBW: Ideal body weight
LG: Lean group
MEN: Maintenance energy requirement
MMS: Muscle mass score
OG: Obese group
OUT: Operational taxonomic units
PCA: Principal component analysis
WLG: Weight loss group
WLP: Weight loss program
